# Patterns of mHealth Engagement and Identification of Facilitators and Barriers to Mobile Health Applications for People Who Use Opioids

**DOI:** 10.3390/ijerph22091396

**Published:** 2025-09-06

**Authors:** Lauren Dayton, Haley Bonneau, Grace Yi, Melissa Davey-Rothwell, Carl Latkin

**Affiliations:** Johns Hopkins Bloomberg School of Public Health, Johns Hopkins University, Baltimore, MD 21205, USA; hbonnea1@jhu.edu (H.B.); gyi1@jhu.edu (G.Y.); mdavey1@jhu.edu (M.D.-R.); carl_latkin@jhu.edu (C.L.)

**Keywords:** mHealth, people who use opioids, app engagement

## Abstract

Mobile health (mHealth) applications represent low-cost, scalable interventions with broad reach and are valuable for people who use opioids (PWUO), a population that often experiences low retention in traditional research studies. This study examines engagement patterns with the OASIS app, an mHealth app prompting PWUO in Baltimore, Maryland, to report daily on substance use locations and overdose prevention behaviors over 14 days. Between December 2022 and September 2023, 210 PWUO participated. Engagement was measured by survey completion, with high engagement defined as completing at least 75% of surveys. The median number of surveys completed was 9.0 (mean: 7.57), and 29.4% of participants demonstrated high engagement. Logistic regression models showed that residential stability was significantly associated with higher engagement (adjusted odds ratio [aOR]: 4.90; 95% CI: 1.35, 17.84), while weekly or more frequent injection drug use was associated with lower engagement (aOR: 0.31; 95% CI: 0.11, 0.89). Mobile device proficiency and other demographics were not significantly associated with engagement, likely due to mandatory in-person training reducing tech barriers. Results indicate that PWUO can meaningfully engage with mHealth apps, especially when supported by structural factors, such as stable housing.

## 1. Introduction

Mobile health (mHealth) applications represent a low-cost, scalable public health intervention that can be widely disseminated. mHealth applications are especially valuable for populations like people who use opioids (PWUO), who may experience low retention rates in traditional studies due to barriers such as unstable housing [1]. In 2023, over 8.3 million adults in the US reported past-year opioid misuse [2], and approximately 100,000 people died from drug overdose [3], highlighting an urgent need to identify effective methods for delivering public health interventions to PWUO. mHealth presents a promising approach, as research indicates high mobile phone ownership among PWUO. For instance, a 2019 study among people who inject drugs in New Haven, Connecticut, found that 68% of participants owned a smartphone and endorsed a strong interest in app-based services [4]. A 2016 study of people who inject drugs in Fresno County, California, similarly found that 77% of participants owned a mobile phone, with 67% having access to a free phone [5]. Although there is high phone ownership [4,5], structural vulnerabilities such as housing instability and drug dependence may impact mHealth engagement. Systematic reviews of smartphone apps among people who misuse substances have largely focused on people with alcohol addiction [6,7], and there is a limited understanding of how PWUO engage with mHealth applications over time. This study aims to describe how engagement in a mHealth application changes over time and to identify barriers and facilitators of high engagement. Specifically, the study examines an mHealth application in which PWUO report daily on their substance use locations and harm reduction behaviors at drug use settings over a 14-day period in Baltimore City, Maryland. The findings from this study will help inform the design of future public health interventions by identifying factors that influence higher mHealth application engagement among PWUO.

mHealth applications targeting PWUO have addressed a range of health outcomes, including medication adherence, withdrawal, harm reduction behaviors, service connection, substance use documentation, and overdose prevention [8,9,10,11,12,13,14]. One such app, “Populi Needle Exchange Point Finder,” was developed to provide health tips and link people who inject drugs (PWID) to needle exchange services using geolocation [8]. Participants found the app highly appealing and useful in increasing access to injection materials. In addition, Guarino and colleagues evaluated the usability of a mobile intervention for individuals in methadone maintenance [11]. The app, a 12-week “Check-In Program,” was designed to identify and manage triggers of drug use through self-management skills. Feedback indicated that participants found the program useful and user-friendly, and participants demonstrated evidence of increased treatment retention and abstinence from illicit opioids compared to those receiving standard methadone maintenance treatment.

Traditional mHealth applications for PWUO primarily focus on overdose prevention by providing information about naloxone access and offering check-in features to promote drug treatment. However, drug use and its consequences are determined in part by the social context. Overdoses often occur in specific environmental and social contexts, making it critical to understand the characteristics of these settings, including the physical environment, geographic location, and presence and behaviors of other people. Such contextual data are essential for informing overdose prevention strategies, reducing the transmission of bloodborne pathogens, and understanding social and geospatial influences on use patterns. The current study focuses on the OASIS app, a novel mHealth tool designed to assess place-based harm reduction behaviors. Place-based harm reduction represents an emerging dimension of overdose prevention that considers how individuals’ harm reduction practices vary across different settings. The OASIS app collects daily data from participants on the locations where drug use occurs and on engagement in harm reduction behaviors at these locations. The app also provides real-time resources, including the nearest site for obtaining naloxone and COVID-19 testing. While this app includes a novel geospatial approach, the findings related to barriers and facilitators of engagement can offer valuable insights to inform the development and optimization of future mHealth interventions targeting PWUO.

A central component of mHealth effectiveness is high user engagement and low attrition; however, limited research has examined patterns of engagement or identified specific factors that influence users’ continued participation. The limited body of mHealth research among people who use drugs has yielded mixed findings. Some studies have found that mHealth interventions are feasible and effective in this population [1,15,16], while others have reported low compliance rates and engagement challenges [17]. A meta-analysis examining compliance with ecological momentary assessment (EMA) protocols among substance-using populations found substantial variability in response rates across 126 studies, with an overall response rate of 75.06% (95% CI = 72.37%, 77.65%) [17]. However, this estimate may be inflated due to the exclusion of participants who failed to meet specific minimum response thresholds. The meta-analysis found no significant associations between response rates and study characteristics such as prompt frequency, assessment duration, substance type, or method of administration. Notably, substance-dependent individuals had lower response rates than participants who were not dependent on substances. A study by Mackesy-Amiti and Boodram investigating engagement with an EMA study examining mood and injection risk behavior among people who inject drugs in Chicago found that women were more responsive than men, while homelessness was associated with lower responsiveness [1]. Interestingly, the frequency of drug use was not associated with engagement in the mobile health application.

One salient barrier identified through qualitative findings was proficiency with mobile devices. Guarino and colleagues reported that participants experienced difficulty using the on-screen keyboard to input responses, while other participants found the phone itself to be complicated [11]. Similarly, Calvo and colleagues received feedback from participants indicating that they thought certain features, such as downloading an app, might be challenging for users to navigate, potentially affecting the app’s usability [8]. However, as the prevalence of smartphones has increased in the population, technological proficiency may also increase in general. The impact of mobile device proficiency on mHealth remains unclear. This study aims to address that gap by exploring the relationship between mobile device proficiency and user engagement in a mHealth application.

## 2. Materials and Methods

### 2.1. Study Participants

Study participants were recruited from December 2022 to September 2023 as part of the OASIS study, a research project to assess geospatial components of drug use and harm reduction. Study recruitment was conducted through street-based outreach, word-of-mouth, flyers, referrals from community agencies, and contacting participants who had previously participated in Lighthouse studies and expressed interest in future studies. Participants were recruited following the completion of the OASIS baseline survey based on the following inclusion criteria: (1) Age 18 or older, (2) Use heroin, fentanyl, and/or prescription opioids at least two times per week, (3) Do not always use drugs at their house, (4) Own a smartphone and/or computer/tablet, (5) Willing to use an app to enter the cross streets of locations where they use drugs and, (6) Willing to respond to a daily survey prompt on their phone for two weeks. A total of 210 eligible participants completed the training session. Sixteen participants were excluded due to only submitting null or invalid responses for all submissions. Following this data cleaning, 194 participants were included in this analysis. Data were collected through a baseline survey, which was administered during face-to-face interviews, followed by a 14-day period when participants independently completed once daily OASIS app surveys via personal mobile device, tablet, or computer.

### 2.2. OASIS App

The OASIS app was developed by the manuscript authors and included a daily 5–10 min survey assessing locations of drug use, overdose prevention behaviors, and harm reduction resource availability at these locations. Location data were jittered prior to data storage for privacy protection. The data entry period for each participant was 14 days long, and daily app completion was monitored using MySQL Workbench. On the first day of the study period, participants completed an app training session with study staff to learn the app’s functionality and complete the first daily survey. The in-person training session took approximately 30 min per participant but could vary greatly depending on participant’s familiarity with the phone and app usage. Training involved introducing the participant to the app, going through a mock daily survey using the app, and having the participant complete the first day of 14 daily surveys. The training session also reviewed the other features of the app with the participant (i.e., turning on notifications, COVID testing locations, locations to obtain naloxone, signing out of the app, tracking previous entries). The OASIS app was available for download in the app store for both iPhone and Android users, and it was also made available as a webpage-based application accessible by mobile phone, tablet, or computer. Participants were instructed to complete the survey daily at whatever time worked best for them. They could only complete the survey once per day. Participants were prompted daily to complete the app survey, and they had the option of turning on device notifications, which would provide a daily reminder at 5 pm to complete the app survey. Participants could also elect to enroll in text message reminders, which would provide a daily text at 5 pm. If two consecutive days elapsed without an entry, those participants were contacted by phone or text message to remind them to complete the daily survey and address any barriers to survey completion. Participants were compensated for each day that they completed a survey. Partial responses were not compensated or included in data analysis. Study participants were compensated $40 for completing the baseline survey, $30 for completing the app training session, and $3 for each daily app survey entry during the 14-day study period.

### 2.3. Measures

#### 2.3.1. Demographics

Demographic questions included age, sex, income, employment, race, education, homelessness in the past 6 months, residential stability, and depression. Age in years was collected as a continuous variable and recategorized into groups of “18–44,” “45–64,” and “65 and older” for analysis. Sex included “Male” and “Female” response options. Monthly household income was dichotomized into “Less than $1000” and “Greater or equal to $1000” categories. The response categories for employment were “Employed full time,” “Employed part time,” “Unemployed,” “Retired,” “Student,” and “Disabled/Unable to work.” For analysis, these categories were collapsed into “Employed (full time and part time)” and “Unemployed (unemployed, retired, student, and disabled/unable to work).” The response categories for race were “African American/Black,” “White,” “Asian,” and “Other.” Due to few responses in the “Asian” and “Other” categories, these were collapsed into a single “Other” category. The response categories for education were “Grade 11 or less,” “Grade 12 or GED,” “Some college, Associate’s Degree, or Technical degree,” and “Bachelor’s degree or higher.” Due to the few responses in the “Bachelor’s Degree or Greater” category, education was dichotomized into “High School Diploma/GED or less” and “Greater than High School Diploma/GED.” Residential stability was dichotomized as “No,” meaning that the participant lives in a shelter/on the street or more than two places a week, and “Yes,” defined as not living in a shelter/on the street or more than two different places a week. Depression scores (Center for Epidemiologic Studies Depression Scale [CES-D-10] score) were dichotomized as “Less than 10,”, indicating no depressive symptoms, and “Greater than 10”, defined as having depressive symptoms [18].

#### 2.3.2. Drug Use

Drug use frequency variables were assessed through the baseline survey and included frequency of opioid (heroin, fentanyl, and/or nonprescription drug) use in the past 3 months and injection drug use in the past 3 months. Frequency of opioid use in the past 3 months was categorized as “Less than weekly” and “Weekly or more often.” Injection drug use in the past three months was dichotomized into “Less than weekly” and “Weekly or more often” responses.

#### 2.3.3. Cellphone Type

The response options for the type of smartphone owned were “Android,” “iPhone,” “Other,” and “Does not own smartphone.” Due to a small number of responses for “Other” and “Does not own a smartphone,” these were collapsed into a single category.

#### 2.3.4. Adapted Mobile Device Proficiency Scale

To assess mobile device proficiency, we adapted a scale developed by Roque and Boot, which was designed to assess mobile device proficiency in older adults using self-report [19]. Questions were adapted for relevance to current cellphone features and to promote parsimony. Study questions were modified based on consultation with experts in mobile health interventions, discussion with community members who use drugs, and participant feedback. The Adapted Mobile Device Proficiency scale included 15 questions assessing proficiency with components of mobile phone use with “yes” and “no” response options. The questions included: (1) Used a touchscreen, (2) Used the onscreen keyboard to type, (3) Connected to WiFi, (4) Sent text messages, (5) Sent pictures by text message, (6) Found information about my hobbies and interests on the internet, (7) Found health information on the internet, (8) Downloaded apps (e.g., games) using the Apple App Store or Google Play, (9) Listened to music, (10) Used an app (e.g., Google Maps, games), (11) Updated an app, (12) Deleted an app, (13) Used video-messaging (e.g., Skype, Google Hangout, Zoom), (14) Set up a password to lock/unlock the device, and (15) Erased all internet browsing history and temporary files. To assess mobile device proficiency, we examined the sum score on the Adapted Mobile Device Proficiency Scale, with a lower score indicating less proficiency with mobile devices and a higher score indicating greater proficiency. The scale has minimum and maximum values of 0 and 15, respectively, with a Cronbach’s alpha of 0.804.

### 2.4. Analysis

Patterns of engagement in the OASIS app were assessed using the total number of surveys completed over the 14-day study period. Engagement patterns over time were visualized to examine engagement dynamics. To assess facilitators and barriers of high engagement in the OASIS app, the number of app surveys completed was dichotomized into “Greater than 75%,” meaning that 11 or more out of the 14 total surveys were completed, and “Less than 75%,” meaning that 10 or fewer surveys were completed. Univariate logistic regression models were used to assess factors associated with high engagement in the OASIS App. Additionally, a multivariate logistic regression model adjusted for confounding variables.

## 3. Results

Table 1 displays demographic characteristics. The majority of participants were between the ages of 45–64 (61%), 34% were between 18 and 44, and 5% were 65 or older. About half the participants were male (56%), and the majority identified as African American/Black (72%). Most were unemployed (93%) and had a household income of less than $1000 (67%). Many of the participants were residentially stable (84%), and 33% had greater educational attainment than a high school diploma/GED.

**Figure 1:**
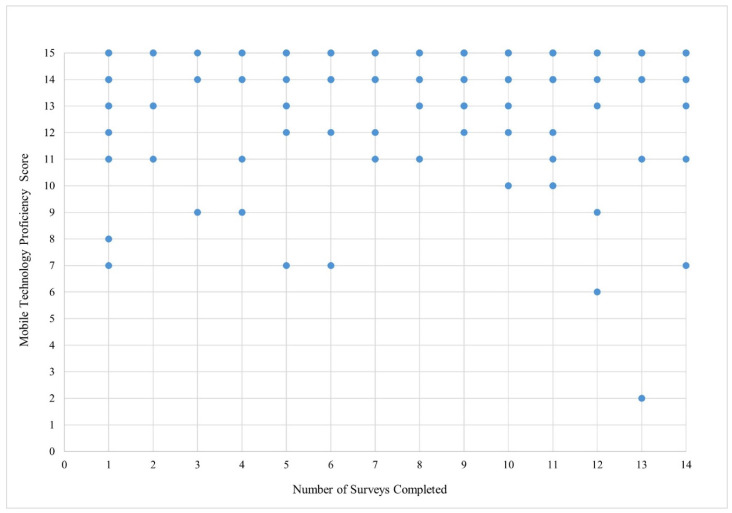
Scatterplot of number of surveys completed and score on the adapted Mobile Technology Proficiency scale (*N* = 194).

Overall, participants scored highly on the adapted Mobile Device Proficiency scale (Table 2, mean: 13.75, SD: 2.05, 95% CI: 13.46, 14.04). Having used a touchscreen was the highest endorsed variable (99.49%, Table 2). The least frequently reported item was erasing all internet browsing history and temporary files (65.45%). The total score on the adapted Mobile Device Proficiency Scale was not significantly associated with engagement in the OASIS App in both univariate and multivariate models (Table 3). Some participants who scored low on the Mobile Device Proficiency scale still showed high engagement in the OASIS app (Figure 1).

**Figure 2:**
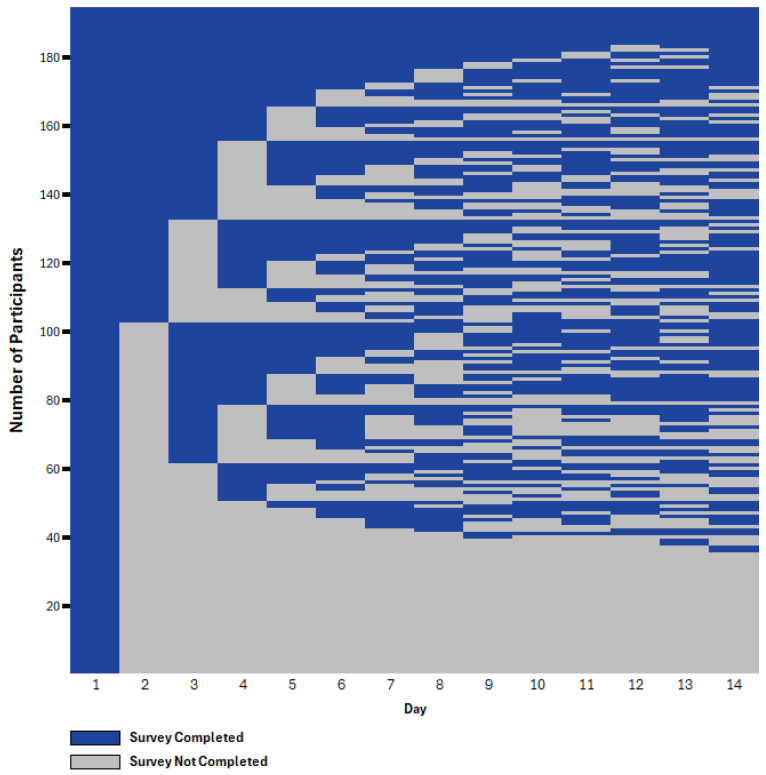
Patterns of OASIS app engagement over 14-day study period (*N* = 194).

**Figure 3:**
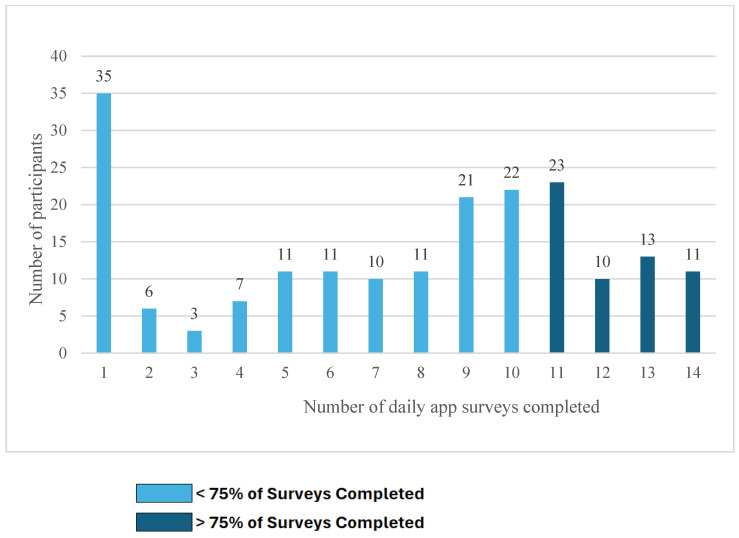
Number of daily OASIS app surveys completed (*N* = 194).

Patterns of engagement in the OASIS app varied substantially as seen in Figure 2. Figure 2 displays a heatmap-style raster plot depicting participant engagement with the OASIS application over the 14-day study period. Each row corresponds to an individual participant, and each column represents a study day. Blue cells denote survey completion, and gray cells denote non-completion. This visual representation highlights individual-level engagement patterns, including consistent participation, intermittent use, and early disengagement. The majority of participants (76.3%) demonstrated varied engagement over time, while 5.7% completed all 14 days of daily surveys, and 18.0% did not complete any surveys after the first in-person training sessions. Figure 3 shows the total number of surveys completed. Overall, 29.4% had high engagement, defined as completing at least 75% of the surveys. The median number of surveys completed was 9.0, with a mean of 7.57 completed. The substantial drop off from 194 entries on day one to 92 on day two may partly reflect transitioning from supervised to unsupervised data entry, as respondents completed the first survey at the study site. There were also reductions in responses from 103 on day three to 94 on day four. However, the majority of participants (94/131) who did not provide responses on days three or four provided responses later in the study.

In both univariate and multivariate analyses, residential stability was associated with increased odds of engagement in the OASIS App (aOR: 4.90, 95% CI: 1.35, 17.84), and greater than weekly injection drug use was associated with decreased engagement (aOR: 0.31, 95% CI: 0.11, 0.89). Depression was significantly associated with reduced app engagement in bivariate (OR: 0.45, 95% CI: 0.24, 0.87) but not in multivariate analyses. Other demographic characteristics such as age, income, and race, as well as type of device, were not significantly associated with app engagement.

Figure 2 displays a heatmap-style raster plot depicting participant engagement with the OASIS application over the 14-day study period. Each row corresponds to an individual participant, and each column represents a study day. Blue cells denote app survey completion, and gray cells denote non-completion.

## 4. Discussion

This study offers a unique contribution to the literature on mHealth interventions by examining the diverse patterns of mHealth engagement among PWUO and identifying factors associated with engagement. It also provides insight into the relationship between mobile device proficiency and engagement in mHealth applications among PWUO. Participants demonstrated varied engagement patterns with the OASIS app, with fluctuating engagement throughout the study period. Despite the challenges faced by PWUO, many were able to engage highly with the OASIS App, which features unique elements such as a geospatial mapping component that identifies locations of drug use. This study aligns with prior research indicating that mHealth interventions are feasible among people who use drugs [1,15,16].

Consistent with the findings of Mackesy-Amiti and Boodram, residential stability was associated with increased engagement with the OASIS App [1]. Individuals with unstable housing often have fluctuating daily routines and environments that may make it difficult to prioritize completion of a daily survey or find a private space to complete the survey. In addition, those experiencing homelessness may have limited access to charging stations. The lack of stable housing may also increase the risk of mobile phones being lost or stolen, further hindering engagement.

Frequent injection drug use was significantly associated with reduced engagement in the OASIS app. This finding contrasts with the work of Mackesy-Amiti and Boodram, who did not find an association between injection drug use and engagement with a health app [1]. Gender did not significantly impact engagement with the OASIS app in this study, whereas Mackesy-Amiti and Boodram identified higher engagement among women compared to men. As their study focused on bivariate associations, it is unclear if gender differences would have persisted in multivariate models. Future research should incorporate both quantitative and qualitative methods to further explore the relationship between gender and engagement in mHealth applications.

Although prior studies reported associations between mobile device proficiency and mHealth engagement [8,11], the adapted Mobile Device Proficiency Scale did not predict engagement with the OASIS app. All items were self-reported, and participants generally endorsed high levels of mobile device proficiency, even after being probed on specific technical features (e.g., updating or deleting an app). This may suggest that participants had high levels of proficiency or over-reported proficiency. It is also possible that basic mobile device proficiency has increased in the general population, and existing scales do not adequately capture this construct. Furthermore, the adapted Mobile Device Proficiency Scale was administered at the end of an hour-long study visit, which could have contributed to participant response fatigue. As the self-report nature of the scale may have caused measurement error, future studies may benefit from using a hands-on assessment of mobile device proficiency, where participants are asked to demonstrate specific skills. Interestingly, some respondents who reported lower mobile device proficiency still demonstrated high engagement with the OASIS app. This may be attributed to the initial training session for the mHealth app, which could have helped mitigate proficiency issues, or high app usability based on design. Additionally, PWUO with lower mobile device proficiency may have relied on support from friends or family to navigate and complete the OASIS app survey.

Future research should further explore patterns of mHealth app engagement. Qualitative interviews and systematic reviews could help identify specific app features that promote high engagement. For instance, apps that incorporate gamification may enhance engagement, particularly among users who prefer this approach, potentially offering a self-sustaining strategy for continued use [20]. Participants from prior studies preferred options to personalize both the frequency and content of messages received regarding the mHealth app [13,14]. The content of a mHealth app may also play a significant role in maintaining interaction. In qualitative feedback on the “Check-In Program,” Guarino and colleagues found that participants reported becoming bored due to the limited content, where only two modules were available, leading to repetitive exercises over the three-month study period [11]. Technical features, such as forgetting logins or the font size, have also been identified as barriers to mHealth app engagement, potentially influencing user interaction over time [11]. The patterns of stops and starts among a large proportion of participants suggest that the reminders may be an important aspect of continued engagement.

Additionally, injection drug use frequency can affect app engagement, as individuals who are frequent injectors may prioritize acquiring drugs and resources to purchase drugs, which may impact their ability or interest in engaging with the mHealth app. Frequent injection drug use may also indicate polydrug use, and the psychoactive properties of these drugs may impact the ability to engage in app usage.

Payment structures and support from research staff can also contribute to engagement levels. In the case of the OASIS app, participants were compensated for each completed entry, and research staff actively checked in with participants to troubleshoot any barriers when participants missed two consecutive days of data collection. Additional research should explore payment structures that may further enhance app engagement.

Several limitations of this study should be noted. First, a limitation of this study is that it was restricted to PWUO who had access to a phone. While this study and previous studies have identified high phone ownership among PWUO, study findings may not be generalizable to PWUO who do not currently have a phone. The study was also limited based on the enrollment criteria, excluding PWUO who only use drugs at their house, which may limit the generalizability of the findings to all PWUO. Additionally, participants may have been unable to engage on certain days due to technical issues (e.g., forgot password, app malfunctions). Though study staff were available to resolve any issues, technical difficulties still may have contributed to disengagement with the app.

## 5. Conclusions

The large sample size and rigorous documentation of daily engagement provide valuable insights into the patterns of mHealth engagement among PWUO. Overall, PWUO demonstrated high engagement with the app, although the majority of participants demonstrated variable engagement patterns. Residential stability and frequent injection drug use emerged as key factors associated with app engagement, whereas mobile device proficiency was not predictive, potentially due to high baseline levels of mobile device proficiency. Future research may also benefit from closer examination of the association of gender and app engagement, as well as continued development of ways to more accurately capture mobile device proficiency using hands-on assessments. Mandatory in-person training sessions and reminder check-ins for incomplete daily entries may have helped address mobile device proficiency problems and improve engagement. These strategies should be considered when developing an mHealth application for PWUO.

## Figures and Tables

**Table 1:** OASIS App Participant Demographics (*N* = 194).

Variable	*n* (%)
Age	
18–44	65 (33.51)
45–64	119 (61.34)
65 and older	10 (5.15)
Sex	
Male	109 (56.19)
Female	85 (43.81)
Income	
Less than $1000	134 (69.07)
Greater or equal to $1000	60 (30.93)
Employment	
Employed	14 (7.22)
Unemployed	180 (92.78)
Race	
African American/Black	139 (71.65)
White	44 (22.68)
Other	11 (5.67)
Education	
High School Diploma/GED or less	131 (67.53)
More than High School Diploma/GED	63 (32.47)
Residential Stability	
Not residentially stable	31 (15.98)
Residentially stable	163 (84.02)
Opioid Use	
Less than weekly	15 (7.73)
Weekly or more	179 (92.27)
CES-D-10 Score	
Less than 10	58 (29.90)
10 or more	136 (70.10)
Type of Phone	
Android	169 (87.11)
iPhone	14 (7.22)
Other/Does Not Own Smart Phone	11 (5.67)
Mobile Device Proficiency Score *	13.75 ± 2.05

* mean ± standard deviation.

**Table 2:** Adapted Mobile Device Proficiency Scale and response frequency *N* = 194).

Using a Mobile Device, I Have:	No (0) *n* (%)	Yes (1) *n* (%)
1. Used a touchscreen	1 (0.52)	193 (99.48)
2. Used the onscreen keyboard to type	3 (1.55)	191 (98.45)
3. Connected to WiFi	13 (6.70)	181 (93.30)
4. Sent text messages	3 (1.55)	191 (98.45)
5. Sent pictures by text message	16 (8.25)	178 (91.75)
6. Found information about my hobbies and interests on the internet	23 (11.86)	171 (88.14)
7. Found health information on the internet	17 (8.76)	177 (91.24)
8. Downloaded apps (e.g., games) using the Apple App Store or Google Play	12 (6.19)	182 (93.81)
9. Listened to music	2 (1.03)	192 (98.97)
10. Used an app (e.g., Google Maps, games)	7 (3.61)	187 (96.39)
11. Updated an app	17 (8.76)	177 (91.24)
12. Deleted an app	15 (7.73)	179 (92.27)
13. Used video-messaging (e.g., Skype, Google Hangout, Zoom)	35 (18.04)	159 (81.96)
14. Set up a password to lock/unlock the device	12 (6.19)	182 (93.81)
15. Erased all internet browsing history and temporary files	67 (34.54)	127 (65.46)

**Table 3:** Univariate and Multivariate Logistic Regression Models of Factors Associated with High Engagement in Mobile App (*N* = 194).

	Univariate Logistic Regression (OR, 95% CI)	Multivariate Logistic Regression with Mobile Device Proficiency Full Scale (aOR, 95% CI)
Age (Ref: 18–44)	REF	REF
45–64	1.50 (0.75, 3.01)	1.07 (0.42, 2.72)
65 and older	3.33 (0.85, 13.08)	2.12 (0.41, 11.00)
Sex (Ref: Male)	0.82 (0.44, 1.53)	0.90 (0.43, 1.88)
Income (Ref: USD Less than 1 K)	0.82 (0.42, 1.63)	0.63 (0.29, 1.35)
Employment (Ref: Unemployed)	0.38 (0.08, 1.75)	0.32 (0.06, 1.65)
Race (Ref: White)	REF	REF
African American/Black	2.23 (0.96, 5.17)	1.29 (0.41, 4.11)
Other	1.69 (0.36, 7.81)	1.86 (0.34, 10.25)
Greater than a high school education	0.84 (0.43, 1.64)	0.82 (0.38, 1.75)
Residentially Stable	**4.62 (1.35, 15.89)**	**4.90 (1.35, 17.84)**
Greater than weekly Opioid Use	0.60 (0.20, 1.76)	0.62 (0.18, 2.11)
Greater than weekly Injection Drug Use	**0.27 (0.11, 0.67)**	**0.31 (0.11, 0.89)**
CES-D-10 Score = 10 or More (Depressive Symptoms)	**0.45 (0.24, 0.87)**	0.49 (0.24, 1.03)
Currently in Drug Treatment	1.14 (0.57, 2.28)	1.11 (0.50, 2.47)
Type of Smartphone (Ref: Android)	REF	REF
iPhone	0.93 (0.28, 3.09)	0.84 (0.23, 3.09)
Other/Does Not Own Smart Phone	0.51 (0.11, 2.46)	0.46 (0.09, 2.40)
Mobile Technology Proficiency Full Scale	0.96 (0.83, 1.11)	1.04 (0.87, 1.22)

note: Bold: *p*-value < 0.05

## Data Availability

The data presented in this study are available on request from the corresponding author.
